# Renal response and acid-base balance alterations during furosemide administration

**DOI:** 10.1186/cc12353

**Published:** 2013-03-19

**Authors:** L Zazzeron, D Ottolina, E Scotti, M Ferrari, M Stanziano, C Rovati, F Zadek, C Marenghi, L Gattinoni, P Caironi

**Affiliations:** 1 Fondazione IRCCS Ca' Granda - Ospedale Maggiore Policlinico, Milan, Italy

## Introduction

Furosemide is one of the most employed diuretics in the ICU for its ability to induce negative water balance. However, one common side effect is metabolic alkalosis [1]. We aimed to describe the time course of urinary excretion and changes in plasmatic acid-base balance in response to the administration of furosemide.

## Methods

We connected the urinary catheter of 39 ICU patients to a quasi-continuous urine analyzer (Kidney INstant monitorinG^®^), allowing measurement of pH (pHU), sodium, chloride, potassium and ammonium concentrations (Na+U, Cl-U, K+U, NH4+U) every 10 minutes. The study period lasted 3 hours after a single intravenous bolus of furosemide (time 0). In 13 patients receiving two or more administrations over a longer period (46 (26 to 49) hours), according to clinical needs, we reviewed data on fluid therapy, hemodynamics and acid-base balance from the beginning to the end of the observation.

## Results

Ten minutes after furosemide administration, Na+U and Cl-U rose from 65 ± 6 to 140 ± 5 and from 109 ± 6 to 150 ± 5 mEq/l respectively, while K+U fell from 60 ± 5 to 39 ± 4 mEq/l (*P *0.001 for all electrolytes vs. time 0) with a consequent increase in urinary anion gap (AGU = Na+U + Cl-U - K+U). Urinary output increased from 10 (5 to 19) to 53 (29 to 71) ml/10 minutes (*P *0.05). After the first hour Cl-U remained higher than Na+U, which progressively decreased, leading to a reduction in AGU and pHU over time. In parallel, a progressive increment in NH4+U was observed. In patients receiving more than one administration we observed an increase in arterial base excess (1.8 ± 0.8 vs. 5.0 ± 0.6 mmol/l, *P *0.001) and plasmatic strong ion difference (SIDpl) (31 (30 to 33) vs. 35 (34 to 36) mEq/l, *P *= 0.01) during the study period. These changes were due to a decrease in plasmatic Cl^- ^concentration (109.0 ± 1.1 vs. 106.6 ± 0.9 mEq/l, *P *= 0.009). Plasmatic sodium and potassium concentrations did not change. In these patients, considering the total amount of administered fluids and urine, a negative water and chloride balance was observed (-460 ± 403 ml and -48 ± 48 mEq, respectively).

## Conclusion

Furosemide acts immediately after administration, causing a rise in urinary output, Na+U and Cl-U concentrations. Loop-diuretic-induced metabolic alkalosis may be due to an increased urinary chloride loss and the associated increase in SIDpl.
